# Synthesis of New 1,2,3-Triazol-4-yl-quinazoline Nucleoside and Acyclonucleoside Analogues

**DOI:** 10.3390/molecules19033638

**Published:** 2014-03-24

**Authors:** Abdelaaziz Ouahrouch, Moha Taourirte, Joachim W. Engels, Soumaya Benjelloun, Hassan B. Lazrek

**Affiliations:** 1 Laboratory of Bioorganic and Macromolecular Chemistry, Department of Chemistry, Faculty of Sciences and Technology Gueliz (FSTG), BP 549, Marrakech 40000, Morocco; 2 Laboratory of Biomolecular and Medicinal Chemistry, Department of Chemistry, Faculty of Sciences Semlalia, Marrakech 40000, Morocco; 3 Institute for Organic Chemistry and Chemical Biology, Goethe-University Frankfurt am Main, Max-von-Laue-Strasse 7, D-60438 Frankfurt am Main, Germany; 4 Viral Hepatitis Laboratory Pasteur Institute Morocco, 1, Place Louis Pasteur, 20360, Casablanca 20001, Morocco

**Keywords:** quinazoline ribonucleosides, 1,2,3-triazole-acyclonucleosides, Huisgen cycloaddition, quinazolinone alkylation, HCV

## Abstract

In this study, we describe the synthesis of 1,4-disustituted-1,2,3-triazolo-quinazoline ribonucleosides or acyclonucleosides by means of 1,3-dipolar cycloaddition between various *O* or *N*-alkylated propargyl-quinazoline and 1'-azido-2',3',5'-tri-*O*-benzoylribose or activated alkylating agents under microwave conditions. None of the compounds selected showed significant anti-HCV activity *in vitro*.

## 1. Introduction

An estimated 150 million people worldwide are chronically infected with hepatitis C virus (HCV) and have an increased risk of eventually developing liver cirrhosis or liver cancer [1]. We believe that a successful approach to cure HCV in most patients will likely require treatment with a combination of drugs that attacks different mechanisms necessary for replication and survival of HCV. Currently, patients undergo treatment with a combination of pegylated interferon alpha and ribavirin or a virus-specific protease inhibitor like telaprevir or boceprevir [2,3,4].

Heterocyclic structures are the basic elements of many pharmaceuticals, agrochemicals and veterinary products. Quinazolinone derivatives are an important class of these heterocyclic compounds that has been shown to display a broad-range of biological activities, for example, anticancer, diuretic, anti-inflammatory, anti-convulsant and antihypertensive activities [5,6,7]. 

In addition, 1,2,3-triazole nucleosides and carbanucleosides are N-Heterocyclic compounds which have been the subject of considerable research, mainly due to their value in synthetic organic chemistry [8,9,10,11,12,13,14] based on the Sharpless-Meldal modified Huisgen reaction. The classical 1,3-dipolar cycloaddition of azides and alkynes discovered by Huisgen [15] often gives mixtures of regioisomers (1,4- and 1,5-disubstituted triazoles). “Click Chemistry” is a term that was developed by Sharpless and independently by Meldal to illustrate a regioselective 1,3-dipolar cycloaddition using Cu(I) salts as catalyst. The catalyst can be added directly in the form of Cu(I) or Cu(II) salts using reducing agents to form active Cu(I) *in-situ* [16,17]. Cu(I) salts require at least an amine base to form the Cu-acetylide complexes. Many studies have shown that the presence of base under the process conditions provides stability for Cu(I) salts against oxidation. It has been used especially in anhydrous media and also under catalytic conditions [18].

In addition, the combination of two different and independently linked hybrid compounds can display synergy and result in a pharmacological potency greater than the sum of each individual moiety’s potencies. For instance, nucleoside analogues incorporating triazole units are a valuable area of therapeutic research, and some triazole-containing compounds have shown activities against hepatitis and HIV-1 [19,20].

We previously reported the preparation of various 1,2,3-triazole acyclonucleosides from propargylated nucleobases by high temperature and long duration copper-free Huisgen 1,3-dipolar cycloadditiona and the evaluation of the resulting compounds for their HIV activity [21]. Recently, we also reported the preparation of several triazolo-acyclic nucleoside phosphonates using copper(I)-catalyzed Huisgen 1,3-dipolar cycloadditions between azido alkylphosphonates and propargylated nucleobases and the evaluation of the resulting compounds for their HIV and HCV activity [22,23,24,25,26,27].

The poor treatment response, combined with often-severe side effects induced by therapy, highlights the need for improved antiviral drugs with better efficacy and safety profiles. Furthermore, in continuation of our research program centered on click chemistry [28,29], the aim of the present work was to synthesize some new hybrid compounds combining the two heterocycles: quinazolinone and 1,2,3-triazole. The new compounds were also assessed for their anti-HCV activities.

## 2. Results and Discussion

### 2.1. Synthesis of Protected Nucleosides and Acyclonucleosides **6a**–**i**

**Scheme 1:**
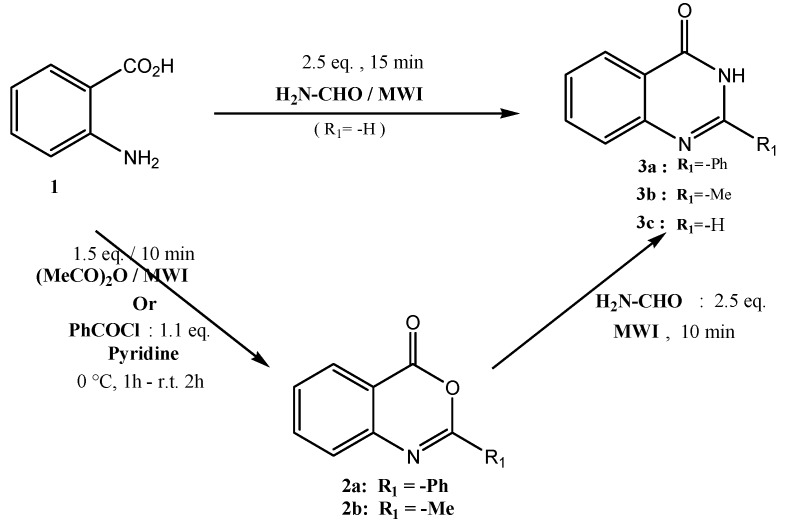
Synthesis of benzoxazinones **2a**,**b**.

Typically, quinazolinone structures were constructed using anthranilic acid or its derivatives via a sequence of acylation and condensation, which requires strong acidic or basic reaction conditions [30,31,32]. The quinazoline derivatives were prepared from anthranilic acid (**1**) in three steps. Initially, the acid was reacted with benzoyl chloride in anhydrous pyridine at 0–5 °C for one hour. Afterwards the reaction mixture was stirred (two hours) at room temperature until 2-phenylbenzoxazinone (**2a**) was formed [33,34]. Alternatively, 2-methylbenzoxazinone (**2b**) was obtained by reaction of anthranilic acid with acetic anhydride using microwave irradiation [35,36]. The benzoxazinones were further treated with formamide under microwave irradiation to obtain the quinazolinones **3a**–**b**. On the other hand, the synthesis of quinazolin-4-one **3c** was achieved by condensing anthranilic acid with 2.5 equivalents of formamide under microwave irradiation [37] (Scheme 1).

**Scheme 2:**
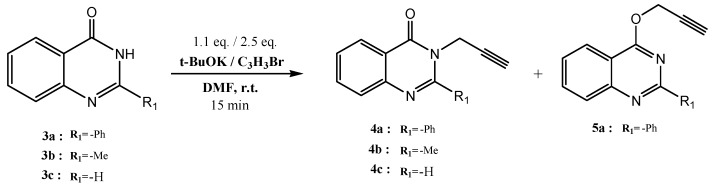
Synthesis of alkylated quinazolines **4a**–**c** and **5a**.

The alkylation of quinazol-4-ones which are substituted in position 2 sometimes leads to two isomers resulting from competing *N*-alkylation and *O*-alkylation and the ratio of these isomers depends on the substituent at position 2. An earlier study confirmed that the substituents and the reaction conditions play a significant role in influencing the ratio of *O*-alkylation *vs.* *N-*alkylation [38]. In this investigation, the quinazolinones **3a**‒**c** were treated with propargyl bromide in the presence of potassium *t*-butoxide. The reaction was carried out using DMF as the solvent [39,40,41] (Scheme 2). The alkylation of 2-methylquinazolin-4-ones **2b** and **2c** leads exclusively to the formation of *N*-propargylated quinazolines, and O-propargylated isomers were not detected. In the case of 2-phenylquinazolin-4-one (**2a**), alkylation preferentially results in O-propargylation, with O and *N*-alkylated products **5a** and **4a** being obtained in a ratio of 58:23. 

**Scheme 3:**
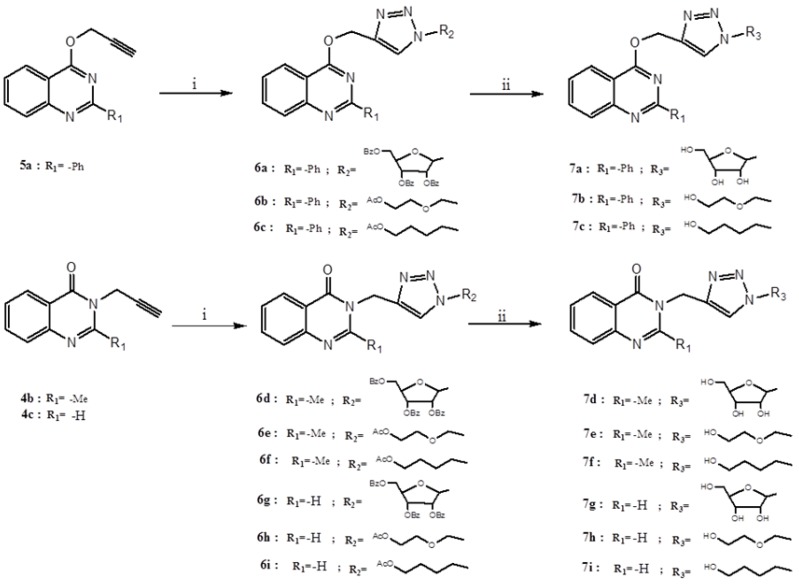
Syntheses of 1,2,3-triazoles-quinazoline **6a**–**i** and **7a**–**i**.

The propargylated quinazolines **4** and **5** were used in the 1,3-dipolar cycloaddition reaction to link 1,2,3-triazole with the quinazoline nucleus to yield compounds **6**. Reaction of the triple bonds of propargylated quinazoline and the azide of sugar and pseudo-sugar was performed under microwave irradiation using Cul as catalyst without solvent (Scheme 3).

*Reaction conditions*: (i) **5a**/**4b**–**c** (1 mmol), alkylazide (2.5 mmol), Et_3_N (1.1 mmol), CuI (0.1 eq), MWI (400W, 2 min); (ii) **6a**,**d**,**g** (1 mmol), NaOMe (1 eq), MeOH, r.t (30 min) or **6b**–**c**,**e**,**f**,**h**,**i** (1 mmol), K_2_CO_3_ (1 eq), MeOH, r.t (15 min).

This protocol (click chemistry) for the formation of the triazole rings is efficient, easy and convenient and typically gives almost quantitative reaction yields [42]. The cycloaddition reaction is drawn in Scheme 3 and the obtained products are tabulated in Table 1.

**Table 1:** Synthesized 1,2,3-triazoloquinazolines **6a**–**i** and **7a**–**i**.

Product	Yield^a^ (%)	Product	Yield ^a^ (%)
**6a**	95	**7a**	98
**6b**	88	**7b**	86
**6c**	93	**7c**	88
**6d**	93	**7d**	97
**6e**	87	**7e**	95
**6f**	86	**7f**	90
**6g**	84	**7g**	98
**6h**	90	**7h**	93
**6i**	89	**7i**	94

^a^ Yields of isolated products.

**Figure 1:**
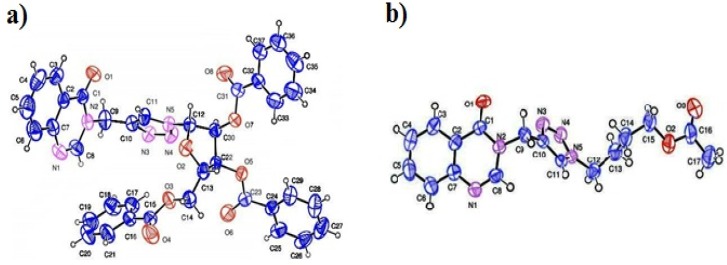
X-ray crystal structures of compounds (**a**) **6g** and (**b**) **6i** with the atom numbering used in the crystallographic analysis.

We characterized the structures of all products by ^1^H-NMR, ^13^C-NMR and mass spectrometry. In addition, the structures of **6g** and **6i** were confirmed by X-ray crystallographic analysis. The structure of crystal (a) illustrates that the anomeric configuration at the C1'-stereocentre has not been affected during the click reaction, the steric effect of benzoyl group in the position 2' and 3' (ribose) directs the reaction towards the β-configuration. The crystal structures of these compounds are shown in Figure 1 [43,44].

### 2.2. Deprotection of Nucleosides and Acyclonucleosides: Preparation of **7a**–**i**

**Figure 2:**
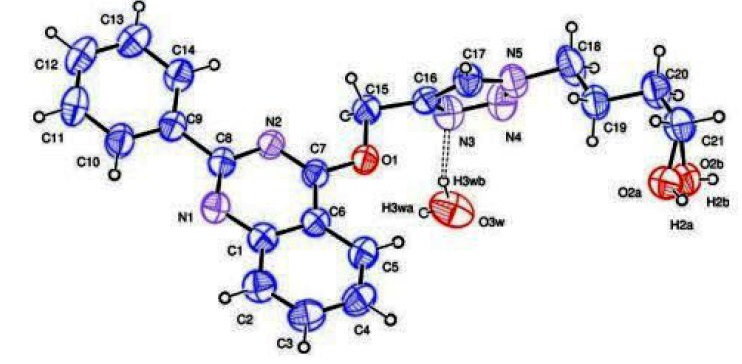
X-ray molecular structure of compound **7c**.

For deprotection base catalyzed methods were employed. Sodium methoxide (NaOMe) in methanol was used for the deprotection of the benzoyl group of compounds **6a**,**d**,**g** [45]. On the other hand, the deprotection of the acetyl group of compounds **6b**,**c**,**e**,**f**,**h**,**i** was carried out using potassium carbonate (K_2_CO_3_) in methanol (Scheme 3 and Table 1). The structure of **7c** was confirmed by X-ray crystallographic analysis. The crystal structure of this compound is shown in Figure 2 [46].

Finally, we were also interested in studying the biological activity of 1,2,3-triazole ribonucleosides 7a‒i. These derivatives were tested *in vitro* to evaluate their anti- HCV activity. None of the new compounds were found to inhibit HCV replication *in vitro* (Table 2).

**Table 2:** Anti-HCV activity of compounds **7a**–**i**.

Compound	CC_50_ (µM) ^a^	IC_50_ (µM) ^b^	SI ^c^
7a	≥50 µM	>10	5
7b	≥100 µM	>10	10
7c	≥50 µM	>10	5
7d	≥100 µM	>10	10
7e	≥50 µM	>10	5
7f	≥50 µM	>10	5
7g	≥100 µM	>10	10
7h	≥50 µM	>10	5
7i	≥50 µM	>10	5
2CmeCyt	>300	1.5	200
aIFNB2	>10,000 ^d^	1.5 ^d^	6667

^a^ CC_50_ Concentrations of compound required for 50% extinction of Huh 5.2 cells; ^b^ IC_50_ Concentrations of compound achieving 50% inhibition of the replicon system; ^c^ SI selectivity index = CC_50_/IC_50_; ^d^ interferon reported as IU/mL.

Antiviral activity was assessed in a 3-day cell culture assay using the HCV-replicon-containing cell line, AVA5 (genotype 1b, CON1) (provided to GUMC by Apath, Inc., Brooklyn, NY, USA) as previously described [47].

## 3. Experimental

### 3.1. General

^1^H- and ^13^C-NMR spectra were recorded in CDCl_3_ or DMSO-*d*_6_ on a Bruker 300 MHz instrument using SiMe_4_ as internal standard. Chemical shifts are given in ppm and coupling constants *(J)* in MHz (br, broad; m, multiplet; t, triplet; d, doublet; and s, singlet). Mass spectra were obtained using ESI/MS and MALDI-TOF-MS. Reactions were carried out in a microwave oven Model AVM510/WP/WH. The reactions were controlled by thin layer chromatography (TLC) on precoated silica gel 60 F254 (Merck, Darmstadt, Germany); UV light was used for visualization of the spots. All products were purified by column chromatography on silica gel (100–200 mesh) Merck.

### 3.2. Synthesis of 2-Substituted Quinazolinones

#### 3.2.1. Preparation of 2-Phenylquinazolin-4-one (**3a**) [36]

Initially, anthranilic acid (**1**, 2 g, 14.5 mmol) was dissolved in dry pyridine (20 mL). Then benzoyl chloride (1.1 equiv) was added dropwise at 0 °C. The reaction mixture was maintained at 0 °C for 1 h and then allowed to stir at room temperature for 2 h, during which time a solid product precipitated out. The mixture was neutralized using a saturated solution of sodium bicarbonate. The pale yellow solid was filtered and washed with water. The product, 2-phenylbenzo[d][1,3]-oxazin-4-one (**2a**) thus obtained was reacted with formamide under microwave irradiation to obtain **3a**. The crude compound obtained was crystallized in ethanol; Mp 225–226 °C; Yield 83%; Rf 0.5; eluent CH_2_Cl_2_/MeOH = 99/1, *v/v*; ^1^H-NMR (CDCl_3_): δ 7.61 (m, 4H, H-Aromatic), 7.83 (m, 1H, H-Aromatic), 8.01 (m, 1H, H-Aromatic), 8.24 (m, 3H, H-Aromatic), 11.9 (s, 1H, N-H). ^13^C-NMR (CDCl_3_): δ 120.81 (C-Aromatic), 126.67, 127.46, 127.85, 128.01, 129.90, 130.11, 131.66, 132.81, 134.93 (CH-Aromatic), 129.04, 149.54, 151.81 (C-Aromatic), 164.04 (CO).

#### 3.2.2. Preparation of 2-Methylquinazolin-4-one (**3b**) [38]

A mixture of anthranilic acid (**1**, 500 mg, 3.64 mmol) and acetic anhydride (1.5 eq) was reacted under microwave irradiation at 400W until total conversion (10 min). After this first step, 2-methylquinazolin-4-one (**3b**) was obtained by addition of formamide (2.5 equiv) to the reaction mixture and irradiated by microwave for 10 min. After cooling to room temperature, the solid obtained was recrystallized from ethanol; Mp 238–240 °C; Yield 94%; R_f_ 0.65; eluent CH_2_Cl_2_/MeOH = 99/1, *v/v*; ^1^H-NMR (CDCl_3_) δ 2.34 (s, 3H, CH_3_-Aromatic), 7.34–8.02 (m, 4H, H- Aromatic), 12.12 (br s, 1H, N-H). ^13^C-NMR (CDCl_3_) δ 39.16 (-CH_3_), 123.31 (C-Aromatic), 125.73, 126.64, 127.15, 134.63 (CH-Aromatic), 156.02, 154.16 (C-Aromatic), 161.63 (CO).

#### 3.2.3. Preparation of Quinazolin-4-one (**3c**) [38]

Anthranilic acid (**1**, 500 mg, 3.64 mmol) and formamide (2.5 equiv) were mixed and irradiated at 400 W for 15 min, the reaction was monitored by TLC. The reaction mixture was cooled to give the crude compound **3c** which was crystallized from ethanol; Mp 217–219 °C; Yield 95%; R_f_ 0.63 (CH_2_Cl_2_); eluent CH_2_Cl_2_; ^1^H-NMR (CDCl_3_) δ (ppm): 7.49 (m, 1H, H-Aromatic), 7.64 (d, 1H, *J =* 8.2 Hz, H-Aromatic), 7.78 (m, 1H, H-Aromatic), 8.07 (s, 1H, H-Aromatic), 8.10 (d, 1H, *J =* 8.2 Hz, H-Aromatic), 12.29 (br s, 1H, NH). ^13^C-NMR (CDCl_3_) δ(ppm): 121.72 (C-Aromatic), 125.46, 126.88, 127.39, 133.98, 145.66 (CH-Aromatic), 147.31 (C-Aromatic), 161.63 (CO).

### 3.3. Synthesis of Propargylated Quinazolines

The appropriate quinazolin-4-one **3a**–**c** (2 mmol) was dissolved in dry DMF (2.5 mL); KO*t*-Bu (1.1 equiv) was added. The mixture was stirred for 15 min at room temperature. Afterwards, propargyl bromide (2.5 mmol) was added dropwise to the mixture. The reaction was performed for 15 min at room temperature. The reaction mixture was diluted with water (10 mL) and extracted with ethyl acetate (2 × 20 mL); the organic phase was dried over Na_2_SO_4_ and evaporated under vacuum. The crude products were purified by column chromatography using CH_2_Cl_2_/MeOH = 99:1, *v/v* as eluent.

*2-Phenyl-4-(prop-2-ynyloxy)quinazoline* (5**a**). Yield 58%; R_f_ 0.80; eluent CH_2_Cl_2_; ^1^H-NMR (CDCl_3_) δ 2.09 (s, 1H, CCH), 5.28 (s, 2H, -CH_2_-N), 7.49–8.60 (m, 9H, H-quinazoline, H-Aromatic). ^13^C-NMR (CDCl_3_) δ 29.93 (-CH_2_-), 53.26 (CH-alkyne), 75.59 (C-alkyne), 121.17 (C-Aromatic) 126.17–135.07 (CH-Aromatic), 128.94, 148.86, 152.19 (C-Aromatic), 162.13 (CO).

*2-Methyl-3-(prop-2-ynyl)quinazolin-4-one* (**4b**). Yield 76%; R_f_ 0.75; eluent CH_2_Cl_2_; ^1^H-NMR (CDCl_3_) δ 2.32 (s, 1H, CCH), 2.76 (s, 3H, CH_3_-Aromatic), 4.94 (s, 2H, -CH_2_-N), 7.48–8.27 (m, 9H, H-quinazoline). ^13^C-NMR (CDCl_3_) δ 22.81 (-CH_3_), 33.13 (-CH_2_-), 72.66 (CH-alkyne), 77.06 (C-alkyne), 120.21(C-Aromatic), 126.71, 134.63, 143.14, 145.06 (CH-Aromatic), 147.10, 153.72 (C-Aromatic), 161.32 (CO).

*3-(Prop-2-ynyl)quinazolin-4-one* (**4c**). Yield 80%; R_f_ 0.40; eluent CH_2_Cl_2_; ^1^H-NMR (CDCl_3_) δ 2.50 (s, 1H, CCH), 4.83 (s, 2H, -CH_2_-N), 7.47–8.30 (m, 9H, H-quinazoline). ^13^C-NMR (CDCl_3_) δ (ppm): 29.08 (-CH_2_-), 71.58 (CH-alkyne), 77.33 (C-alkyne), 120.18 (C-Aromatic), 126.43, 134.26, 142.98, 145.32 (CH-Aromatic), 146.89, 151.62 (C-Aromatic), 162.06 (CO).

### 3.4. Synthesis of 1,2,3-Triazol-4-yl substituted O-, N-Quinazolines

Propargylated quinazolines **5a**/**4b**–**c** (1 mmol), alkyl azide (sugar or pseudosugar, 2.5 mmol) and Et_3_N (1.1 mmol) were mixed with CuI (0.1 equiv). For homogenization, the reaction mixture was dissolved in dry MeCN and stirred for 5 min. Then, the solvent was removed under vacuum. The mixture was subjected to microwave irradiation at the power level 400 W for 2 min. The residue was purified on silica gel using CH_2_Cl_2_ as eluent.

*4-((1-(2,3,5-Tri-O-benzoyl-β-d-ribofuranos-1-yl)1H-1,2,3-triazol-4-yl)methoxy)-2-phenylquinazoline* (**6a**). Yield 95%; R_f_ 0.63; eluent CH_2_Cl_2_; ^1^H-NMR (CD Cl_3_) δ 4.48 (m, 1H, H_5'_), 4.64 (m, 1H,H_5'_), 4.75 (m, 1H,H_4'_), 5.70 (s, 2H, -CH_2_-), 6.05 (dd, 1H, *J =* 8.2 Hz, H_3'_), 6.21 (dd, 1H, *J* = 8.2 Hz, H_2'_), 6.32 (d, 1H, H_1'_), 7.14–7.44 (m, 10H, H-Aromatic) 7.65–7,81 (m, 12H, H-Aromatic), 7.62 (s, 1H, H-Triazole), 8.83–8.88 (m, 2H, H-Aromatic). ^13^C-NMR (CDCl_3_) δ 57.21, 61.07 (CH_2_), 69.13(C5'), 72.81(C2'), 78.72(C3'), 87.72 (C4'), 112.58(C1'), 123.30, 126.59, 127.91, 128.44–133.88 (CH-Aromatic), 135.68–158.34 (C-Aromatic), 163.12, 164.45 (CO). ESI-MS(M+H)^+^, *m/z* calcd for C_43_H_33_N_5_O_8_ 748.75, found 748.90.

*2-((4-((2-Phenylquinazolin-4-yloxy)methyl)-1,2,3-triazol-1-yl)methoxy)ethylacetate* (**6b**). Yield 88; R_f_ 0.62; eluent CH_2_Cl_2_; ^1^H-NMR (CDCl_3_) δ 1.80 (s, 3H, -CO-CH_3_), 3.63 (t, 2H, -CH_2_-O), 4.35 (t, 2H, -CH_2_-O-CO), 4.51 (s, 2H, N-CH_2_-Triazole), 5.88 (s, 2H, O-CH_2_-Triazole), 7.45 (m, 4H, H-Aromatic), 7.73 (m, 2H, H-Aromatic), 7.84 (s, 1H, H-Triazole), 8.11 (dd, 1H, H-Aromatic), 8.54(dd, 2H, H-Aromatic). ^13^C-NMR (CDCl_3_) δ 20.42(CH3), 49.13 (CH_2_-quinazoline), 59.78, 62.62 (-OCH_2_CH_2_O-), 67.69 (-O-CH_2_-N<), 123.51–133.80 (-CH-Aromatic), 142.13,146.46, 152.22 (C-Aromatic), 162.43, (CO-Aromatic),172.12 (CO-Ester). ESI-MS(M+H)^+^, *m/z* calcd for C_22_H_21_N_5_O_4_ 420.43, found 420.40.

*4-(4-((2-Phenylquinazolin-4-yloxy)methyl)-1,2,3-triazol-1-yl)butylacetate* (**6c**). Yield 83%; R_f_ 0.68; eluent CH_2_Cl_2_; ^1^H-NMR (CDCl_3_) δ 1.56 (s, 3H, -CO-CH_3_), 1.92 (m, 4H, -CH_2_-CH_2_-), 3.97 (t, 2H, *J =* 6.3 Hz,-CH_2_-N(Triazole)), 4.29 (t, 2H, *J =* 7.2 Hz, -CH_2_-O-CO), 5.85 (s, 2H, O-CH_2_-C(Triazole)), 7.46 (m, 4H, H-Aromatic), 7.72 (m, 2H, H-Aromatic), 7.93 (s, 1H, H-Triazole), 8.10 (dd, 1H, H-Aromatic), 8.56 (dd, 2H, H-Aromatic).^13^C-NMR (CDCl_3_) δ 18.61(CH3), 23.38, 24.71 (-CH_2_-CH_2-_) 47.62(-CH_2_-quinazoline), 57.67 (CH_2_-N(Triazole)), 61.06 (CH_2_-O), 123.59–133.77 (CH-Aromatic), 142.13, 146.46, 152.22 (C-Aromatic),163.86, (CO-Aromatic), 168.70 (CO-Ester). ESI-MS (M+H)^+^, *m/z* calcd for C_23_H_23_N_5_O_3_ 418.46, found 418.60.

*3-((1-(2,3,5-Tri-O-benzoyl-β-D-ribofuranos-1-yl)-1,2,3-triazol-4-yl)methyl)-2-methylquinazolin-4-one* (**6d**). Yield 83%; R_f_ 0.60; eluent CH_2_Cl_2_; ^1^H-NMR (CDCl_3_) δ 2.76 (s, 3H, -CH_3_), 4.54 (m, 1H, H_5__'_), 4.65 (m, 1H, H_5__'_), 4.81 (m, 1H, H_4__'_), 5.23 (s, 2H, -CH_2_-), 6.10 (dd, 1H, *J =* 8.6 Hz, H_3__'_), 6.19 (dd, 1H, *J =* 8.6 Hz, H_2__'_), 6.31(d, 1H, H_1__'_), 7.23–7,40(m, 13H, H-Aromatic), 7.57(s, 1H, H-Triazole), 8.62–7.86 (m, 5H, H-Aromatic), 7.88 (m, 1H, H-Aromatic).^13^C-NMR (CDCl_3_) δ 23.67(CH_3_), 39.45, 63.75(CH_2_), 71.70 (C5'), 75.22 (C2'), 81.13 (C3'), 90.34 (C4'), 117.84 (C1'), 124.07–134.35 (CH-Aromatic), 140.73–159.53 (C-Aromatic), 162.51, 163.58 (CO). ESI-MS(M+H)^+^, *m/z* calcd for C_38_H_31_N_5_O_8_ 686,68, found 686.50.

*2-((4-((2-Methyl-4-oxoquinazolin-3-yl)methyl)-1,2,3-triazol-1-yl) methoxy) ethylacetate* (**6e**). Yield 87%; R_f_ 0.59; eluent CH_2_Cl_2_; ^1^H-NMR (CDCl_3_) δ 1.9(s, 3H, -CO-CH_3_), 2.84 (s, 3H, Aromatic-CH_3_), 3.64 (t, 2H, -CH_2_-O), 4.35 (t, 2H, -CH_2_-O-CO), 4.52 (s, 2H, N-CH_2_-Triazole), 5.33 (s, 2H, O-CH_2_-Triazole), 7.34–7.55 (m, 3H, H-Aromatic), 7.78 (s, 1H, H-Triazole), 8.13 (m, 1H, H-Aromatic). ^13^C-NMR (CDCl_3_) δ 20.23, 23.86 (CH3), 39.59 (CH_2_-quinazoline), 49.04, 62.16 (OCH_2_CH_2_O), 78.77 (O-CH_2_-N), 120.24–134.34 (CH-Aromatic), 143.00, 147.35, 154.36 (C-Aromatic), 162.01 (CO-Aromatic), 170.25 (CO-Ester). ESI-MS(M+H)^+^, *m/z* calcd for C_17_H_19_N_5_O_4_ 358.36, found 358.40.

*4-(4-((2-Methyl-4-oxoquinazolin-3-yl)methyl)-1,2,3-triazol-1-yl)butylacetate* (**6f**). Yield 86%; R_f_ 0.60; eluent CH_2_Cl_2_; ^1^H-NMR (CDCl_3_) δ 1.55 (s, 3H, -CO-CH_3_), 1.90 (m, 4H,-CH_2_-CH_2_-), 2.83 (s, 3H, Aromatic-CH_3_), 3.99 (t, 2H, *J =* 6.2Hz, -CH_2_-N(Triazole)), 4.27 (t, 2H, *J =* 7.2Hz, -CH_2_-O-CO), 5.29 (s, 2H, O-CH_2_-C(Triazole)), 7.20–7,52 (m, 3H, H-Aromatic), 7.63 (s, 1H, H-Triazole), 8.17 (m, 1H, H-Aromatic). ^13^C-NMR (CDCl_3_) δ (ppm): 20.88, 23.74 (CH_3_), 25.67, 26.87 (CH_2_-CH_2_) 39.72 (CH_2_-quinazoline), 49.91 (CH_2_-N(Triazole)), 63.32 (CH_2_-O), 123.89–134.46(CH-Aromatic), 142.93, 147.42, 154.44 (C-Aromatic), 162.71, (CO-Aromatic), 168.70 (CO-Ester). ESI-MS(M+H)^+^, *m/z* calcd for C_18_H_21_N_5_O_3_ 356.39, found 356.40.

*3-((1-(2,3,5-Tri-O-benzoyl-β-d-ribofuranos-1-yl)-1,2,3-triazol-4-yl)methyl)quinazolin-4-one* (**6g**). Yield 84%; R_f_ 0.60; eluent CH_2_Cl_2_; ^1^H-NMR (CDCl_3_) δ (ppm): 4.68 (m, 1H, H_5__'_), 4.75 (m, 1H, H_5__'_), 4.91 (m, 1H, H_4__'_), 5.30 (s, 2H, -CH_2_-), 6.16 (dd, 1H, *J =* 7.8 Hz, H_3__'_), 6.26 (dd, 1H, *J =* 7.8 Hz, H_2__'_), 6.38(d, 1H, H_1__'_), 7.27–7.40 (m, 13H, H-Aromatic), 7.69 (s, 1H, H-Triazole), 7.87–8.08 (m, 5H, H-Aromatic), 7.30 (m, 1H, H-Aromatic). ^13^C-NMR (CDCl_3_) δ (ppm): 41.46, 63.68 (CH_2_), 71.65 (C5'), 75.20 (C2'), 81.24 (C3'), 90.42 (C4'), 122 (C1'), 123.56–134.36 (CH-Aromatic), 142.46–160.90 (C-Aromatic), 164.98, 166.05 (CO). ESI-MS(M+H)^+^, *m/z* calcd for C_37_H_29_N_5_O_8_ 672.65, found 672.80.

*2-((4-((4-Oxoquinazolin-3-yl)methyl)-1,2,3-triazol-1-yl)methoxy)ethylacetate* (**6h**). Yield 90%; R_f _0.57; eluent CH_2_Cl_2_; ^1^H-NMR (CDCl_3_) δ 1.9 (s, 3H, -CO-CH_3_), 3.64 (t, 2H,-CH_2_-O), 4.35 (t, 2H, -CH_2_-O-CO), 4.52 (s, 2H, N-CH_2_-Triazole), 5.33 (s, 2H, O-CH_2_-Triazole), 7.21-7.42 (m, 3H, H-Aromatic), 7.77 (s, 1H, H-Triazole), 8.18–8.30 (m, 2H, H-Aromatic). ^13^C-NMR (CDCl_3_) δ 20.63 (CH_3_), 41.63 (CH_2_-quinazoline), 49.15, 62.19 (-OCH_2_CH_2_O-), 79.11 (-O-CH_2_-N<), 121.94–134.44 (CH-Aromatic), 142.43, 146.30, 148.14 (C-Aromatic), 160.99 (CO-Aromatic), 170.34 (CO-Ester). ESI-MS (M+H)^+^, *m/z* calcd for C_16_H_17_N_5_O_4_ 344.34, found 344.10.

*4-(4-((4-Oxoquinazolin-3-yl)methyl)-1,2,3-triazol-1-yl)butylacetate* (**6i**). Yield 89%; R_f_ 0.60; eluent CH_2_Cl_2_; ^1^H-NMR (CDCl_3_) δ 1.55 (s, 3H, -CO-CH_3_), 1.90 (m, 4H,-CH_2_-CH_2_-), 3.40 (t, 2H, *J =* 6.3 Hz, -CH_2_-N(Triazole)), 4.28 (t, 2H, *J =* 7.2 Hz, -CH_2_-O-CO), 5.20 (s, 2H, O-CH_2_-C(Triazole)), 7.20 (m, 1H, H-Aromatic), 7.65 (s, H, H-Aromatic, H-Triazole), 8.19–8.28 (m, 2H, H-Aromatic). ^13^C-NMR (CDCl_3_) δ 20.88 (CH_3_), 25.66, 26.87 (CH_2_-CH_2_) 39.72(CH_2_-quinazoline), 49.91 (CH_2_-N(Triazole)), 63.32 (CH_2_-O), 123.89–134.46 (CH-Aromatic), 142.93, 147.42, 154.44 (C-Aromatic), 162.71, (CO-Aromatic), 168.70 (CO-Ester). ESI-MS(M+H)^+^, *m/z* calcd for C_17_H_19_N_5_O_3_ 342.36, found 342.33.

#### 3.4.1. Benzoyl Group Deprotection

The compound (**6a**,**d**,**g**, 1 mmol) was dissolved in dry methanol (2.5 mL). NaOMe (1 eq) was added to the solution with stirring for 30 min at room temperature. The neutralization was performed with AmberliteIR120 hydrogen form. After, filtration and evaporation the residue was purified by silica gel flash column chromatography.

#### 3.4.2. Acetyl Group Deprotection

A solution of (**6b**,**c**,**e**,**f**,**h**,**i**, 1 mmol) in dry methanol (2.5 mL) was treated with 1 eq of K_2_CO_3_. The reaction mixture was stirred at room temperature for 15 min. The deprotected compound was purified by silica gel flash column chromatography.

*4-((1-(β-D-Ribofuranos-1-yl)-1,2,3-triazol-4-yl)methoxy)-2-phenylquinazoline* (**7a**). Yield 98%; R_f_ 0.56; eluent CH_2_Cl_2_/MeOH = 95/5: *v/v*; ^1^H-NMR (DMSO-*d*_6_) δ 2.50 (d, 2H, -CH_2_-), 3.35–3.65 (m, 3H, -OH), 4.18 (m, 1H, H_5'_), 4.42 (m, 1H,H_5'_), 4.91 (m, 1H,H_4'_), 5.88 (s, 2H, -CH_2_-), 5.28 (dd, 1H, *J =* 8.2 Hz, H_3'_), 5.71 (dd, 1H, *J =* 8.2 Hz, H_2'_), 6.01 (d, 1H,H_1'_),. 7.55 (m, 4H, H-Aromatic) 7.97 (m, 2H, H-Aromatic), 8.25 (s, 1H, H-Triazole), 8.58 (m, 3H, H-Aromatic). ^13^C-NMR (DMSO-*d*_6_) δ 59.81 (CH_2_), 61.31 (C5'), 70.36 (C2'), 75.08 (C3'), 85.89 (C4'), 92.10 (C1'), 114.38–130.83 (CH-Aromatic), 134.36–158.82 (C-Aromatic), 165.74 (CO). ESI-MS(M+H)^+^, *m/z* calcd for C_22_H_21_N_5_O_5_ 436.43, found 436.30.

*2-((4-((2-Phenylquinazolin-4-yloxy)methyl)-1,2,3-triazol-1-yl)methoxy)etanol* (**7b**). Yield 86%; R_f_ 0.43; eluent CH_2_Cl_2_/MeOH, 95/5: *v/v*; ^1^H-NMR (DMSO-*d*_6_) δ 3.63 (t, 4H,-CH_2_-O), 4.24 (s, 2H, N-CH_2_-Triazole), 4.65 (t, 1H, -OH), 5.28 (s, 2H, O-CH_2_-Triazole), 7.67 (m, 4H, H-Aromatic), 7.67 (m, 2H, H-Aromatic), 7.96 (s, 1H, H-Triazole), 8.54 (dd, 3H, H-Aromatic). ^13^C-NMR (DMSO-*d*_6_) δ 20.32 (CH_3_), 49.13 (CH_2_-quinazoline), 59.78 (CH_2_OH), 62.62 (OCH_2_), 66.87 (O-CH_2_-N), 124.50–134.20 (CH-Aromatic), 141.7–153.60 (C-Aromatic), 161.32, (CO-Aromatic). ESI-MS(M+H)^+^, *m/z* calcd for C_20_H_19_N_5_O_3_ 378.40, found 378.30.

*4-(4-((2-Phenylquinazolin-4-yloxy)methyl)-1,2,3-triazol-1-yl)butan-1-ol* (**7c**). Yield 88%; R_f_ 0.44; eluent CH_2_Cl_2_/MeOH = 95/5, *v/v*; ^1^H-NMR (DMSO-*d*_6_) δ 1.45–1.86 (m, 4H,-CH_2_-CH_2_-), 2.50 (t, 1H, -OH), 4.38 (s, 2H, -CH_2_OH), 4.39 (t, 2H, *J =* 6.3Hz, -CH2-N(Triazole)), 5.85 (s, 2H, N-CH_2_-Triazole), 7.54 (m, 4H, H-Aromatic), 7.87 (m, 3H, H-Aromatic), 8.32 (s, 1H, H-Triazole), 8.57 (m, 2H, H-Aromatic). ^13^C-NMR (DMSO-*d*_6_) δ 26.56–29.19 (CH_2_-CH_2_) 47.62 (CH_2_-quinazoline), 57.67 (CH_2_-N(Triazole)), 61.06 (CH_2_-O), 114.41–130.80 (CH-Aromatic), 134.30–158.80 (C-Aromatic), 165.47 (CO-Aromatic). ESI-MS(M+H)^+^, *m/z* calcd for C_21_H_23_N_5_O_2_ 376.44, found 376.50.

*3-((1-(β-d-Ribofuranos-1-yl)-1,2,3-triazol-4-yl)methyl)-2-methylquinazolin-4-one* (**7d**). Yield 97%; R_f_ 0.60; eluent CH_2_Cl_2_/MeOH = 95/5, *v/v*; ^1^H-NMR (DMSO-*d*_6_) δ 2.74 (s, 3H, -CH_3_), 3.56–4.22 (m, 3H, -OH), 4.67 (m, 1H, H_5__'_), 4.98 (m, 1H, H_5__'_), 5.22 (m, 1H, H_4__'_), 5.41 (s, 2H, -CH_2_-), 6.10 (dd, 1H, *J =* 8.6 Hz, H_3__'_), 5.62 (dd, 1H, *J =* 8.6 Hz, H_2__'_), 6.01 (d, 1H, H_1__'_), 7.46–7.60 (m, 2H, H-Aromatic), 7.83 (m, 1H, H-Aromatic), 8.11 (s, 1H, H-Triazole), 8.32 (m, 1H, H-Aromatic). ^13^C-NMR (DMSO-*d*_6_) δ(ppm): 23.67 (CH_3_), 58.81 (CH_2_), 61.23 (C5'), 70.28 (C2'), 74.94 (C3'), 85.83 (C4'), 92.03 (C1'), 119.80–126.52 (CH-Aromatic), 134.45–155.00 (C-Aromatic), 160.90 (CO). ESI-MS (M+H)^+^, *m/z* calcd for C_17_H_19_N_5_O_5_ 374.36, found 374.30.

*2-((4-((2-Methyl-4-oxoquinazolin-3-yl)methyl)-1,2,3-triazol-1-yl)methoxy)ethanol* (**7e**). Yield 95%; R_f_ 0.40; eluent CH_2_Cl_2_/MeOH = 95/5, *v/v*; ^1^H-NMR (DMSO-*d*_6_) δ 2.74 (s, 3H, -CH_3_), 3.39 (t, 2H, -CH_2_-O), 3.64 (t, 2H, -CH_2_-O), 4.50 (s, 2H, N-CH_2_-Triazole), 4.63 (t, 1H, -OH), 5.21 (s, 2H, O-CH_2_-Triazole), 7.46–7,65 (m, 2H, H-Aromatic), 7.82 (s, 1H, H-Triazole), 8.07 (m, 2H, H-Aromatic). ^13^C-NMR (DMSO-*d*_6_) δ 22.98 (CH_3_), 40.31 (CH_2_-quinazoline), 59.78 (CH_2_OH), 62.62 (OCH_2_), 80.27 (O-CH_2_-N), 119.83–134.42 (CH-Aromatic), 142.04, 147.03, 155.03 (C-Aromatic), 160.92 (CO-Aromatic). ESI-MS(M+H)^+^, *m/z* calcd for C_15_H_17_N_5_O_3_ 316.33, found 316.40.

*4-(4-((2-Methyl-4-oxoquinazolin-3-yl)methyl)-1,2,3-triazol-1-yl)butan-1-ol* (**7f**). Yield 90%; R_f_ 0.39; eluent CH_2_Cl_2_/MeO = 95/5, *v/v*; ^1^H-NMR (DMSO-*d*_6_) δ (ppm): 1.43–1.88 (m, 4H, -CH_2_-CH_2_-), 2.61 (s,3H, -CH3), 2.72 (t, 1H, -OH), 4.37 (s, 2H, -CH_2_OH), 4.40 (t, 2H, *J =* 6.3 Hz, -CH2-N(Triazole)), 5.56 (s, 2H, N-CH_2_-Triazole), 7.58–7.69 (m, 2H, H-Aromatic), 7.87 (s, 1H, H-Triazole), 8.17(m, 2H, H-Aromatic). ^13^C-NMR (DMSO-*d*_6_) δ 22.96 (CH_3_), 26.48, 29.19 (CH_2_-CH_2_) 40.30 (CH_2_-quinazoline), 49.31 (CH_2_-N(Triazole)), 59.94 (CH_2_-O), 119.82–134.41 (CH-Aromatic), 142.23, 147.03, 155.01 (C-Aromatic), 160.91 (CO-Aromatic). ESI-MS(M+H)^+^, *m/z* calcd for C_16_H_19_N_5_O_2_ 314.35, found 314.30.

*3-((1-(β-D-Ribofuranos-1-yl)-1,2,3-triazol-4-yl)methyl)quinazolin-4-one* (**7g**). Yield 98%; R_f_: 0.62; eluent CH_2_Cl_2_/MeOH = 95/5, *v/v*; ^1^H-NMR (DMSO-*d*_6_) δ 3.52–4.33 (m,3H,-OH), 4.59 (m, 1H, H_5__'_), 4.70 (m, 1H, H_5__'_), 4.88 (m, 1H, H_4__'_), 5.28 (s, 2H, -CH_2_-), 5.66 (dd, 1H, *J =* 7.8 Hz, H_3__'_), 5.73 (dd, 1H, *J =* 7.8 Hz, H_2__'_), 6.20 (d, 1H, H_1__'_),. 7.44–7.58 (m, 3H, H-Aromatic), 7.63 (s, 1H, H-Triazole), 7.92 (m, 2H, H-Aromatic). ^13^C-NMR (75 MHz, DMSO-*d*_6_) δ 40.86 (CH_2_), 61.28 (C5'), 70.31 (C2'), 74.97 (C3'), 85.85 (C4'), 92.02 (C1'), 121.53–132.23 (CH-Aromatic), 134.44, 142.60, 147.83 (C-Aromatic), 159.84 (CO). ESI-MS(M+H)^+^, *m/z* calcd for C_16_H_17_N_5_O_5_ 360.34, found 360.42.

*2-((4-((4-Oxoquinazolin-3-yl)methyl)-1,2,3-triazol-1-yl)methoxy)ethanol* (**7h**). Yield 93%; R_f_: 0.39; eluent CH_2_Cl_2_/MeOH = 95/5, *v/v*; ^1^H-NMR (DMSO-*d*_6_) δ 3.64 (m, 4H, -CH_2_-O), 4.23 (t, 1H, -OH), 4.56 (s, 2H, N-CH_2_-Triazole), 5.40 (s, 2H, O-CH_2_-Triazole), 7.62–7.79 (m, 3H, H-Aromatic), 7.80 (m, 2H, H-Triazole, H-Aromatic), 8.33 (s, 1H, H-Aromatic). ^13^C-NMR (DMSO-*d*_6_) δ 52.20 (CH_2_-quinazoline), 59.70 (CH_2_OH), 70.97 (OCH_2_), 78.21 (O-CH_2_-N), 121.45–134.41 (CH-Aromatic), 142.00, 147.82, 155.12 (C-Aromatic), 159.84 (CO-Aromatic). ESI-MS(M+H)^+^, *m/z* calcd for C_14_H_15_N_5_O_3_ 302.30, found 302.30.

*4-(4-((4-Oxoquinazolin-3-yl)methyl)-1H-1,2,3-triazol-1-yl)butan-1-ol* (**7i**). Yield 94%; R_f_: 0.43; eluent CH_2_Cl_2_/MeOH = 95/5, *v/v*; ^1^H-NMR (CDCl_3_) δ 1.39–1.87 (m, 4H,-CH_2_-CH_2_-), 2.56 (t, 1H, -OH), 4.36 (s, 2H, -CH_2_OH), 4.43 (t, 2H, *J =* 6.3 Hz, -CH_2_-N(Triazole)), 5.32 (s, 2H, N-CH_2_-Triazole), 7.58–7.69 (m, 2H, H-Aromatic), 7.87 (s, 1H, H-Triazole), 8.17 (m, 2H, H-Aromatic). ^13^C-NMR (DMSO-*d*_6_) δ 26.49, 29.18 (CH_2_-CH_2_) 40.94 (CH_2_-quinazoline), 49.30 (CH_2_-N(Triazole)), 59.94 (CH_2_-O), 121.54–127.19 (CH-Aromatic), 142.20, 147.81, 147.815 (C-Aromatic), 159.84, (CO-Aromatic). ESI-MS(M+H)^+^, *m/z* calcd for C_15_H_17_N_5_O_2_ 300.33, found 300.30.

## 4. Conclusions

In summary, the synthesis of a series of 1,2,3-triazole-4-yl-quinazoline derivatives starting from anthranilic acid was performed efficiently using click chemistry under microwave irradiation. None of the compounds selected showed significant anti-HCV activity.
